# Robot-Assisted Uterus-Sparing Repair of a Vesicouterine Fistula 26 Years After Cesarean Section: A Case Report and Review of Surgical Techniques

**DOI:** 10.1007/s00192-025-06304-w

**Published:** 2025-10-08

**Authors:** Fabien Romito, Chahin Achtari

**Affiliations:** DFME, Centre Hospitalier Universitaire Vaudois, Lausanne, Switzerland

**Keywords:** Robot-assisted, Fistula, Menouria, Youssef's syndrome

## Abstract

**Introduction and Hypothesis:**

This video illustrates the surgical repair of a vesicouterine fistula (VUF) via a transperitoneal, robot-assisted, uterus-sparing approach.

**Methods:**

In this video, we present a VUF that occurred after a cesarean section. The patient presented 22 years later with persistent hematuria. She underwent a uterus-sparing, robot-assisted repair via excision of the fistula tract. The uterus and bladder were closed in multiple layers with omental interposition.

**Results:**

The patient tolerated the procedure well. A follow-up cystography confirmed the absence of defects or fistula recurrence. She has remained asymptomatic since the surgery.

**Conclusions:**

Our video demonstrates the feasibility of vesicouterine fistula repair with uterine preservation using an exclusively robot-assisted transperitoneal approach with omental interposition, yielding excellent results.

**Supplementary Information:**

The online version contains supplementary material available at 10.1007/s00192-025-06304-w.

## Introduction

Vesicouterine fistulas (VUFs) are abnormal communications between the uterus and the bladder, representing a rare subset of urogenital fistulas, accounting for 1% to 4% of cases [1–5]. The increasing global rate of cesarean sections has led to a rise in VUF incidence [3]. Clinically, VUFs present most commonly with cyclic hematuria (menouria), vaginal urinary leakage, or secondary infertility [6]. Although spontaneous closure is rare (approximately 5% of cases [1, 4]), most fistulas require surgical intervention.

This video illustrates the robot-assisted, transperitoneal, uterus-sparing repair of a VUF, highlighting a minimally invasive approach with omental interposition, performed 26 years after the index cesarean section.

## Review of Similar Cases and Repair Techniques

The majority of VUFs are diagnosed within months or a few years after cesarean section, with delayed presentations, as in our case, being exceptionally rare [3, 7]. Youssef first described the classic presentation of cyclic hematuria in 1957 [6], whereas others reported variable intervals between cesarean delivery and fistula onset, ranging from months to over a decade [2, 5, 7]. Our case stands out, with a 26-year latency, one of the longest reported in the literature.

Surgical approaches to VUF repair vary:

- Transvaginal approach: suitable for small, low-lying fistulas but limited by access and visualization challenges [8].
- Transvesical–retroperitoneal approach (O'Connor technique): historically considered a standard for complex fistulas, allowing layered closure and omental interposition [9].
- Transperitoneal approach: offers optimal exposure, especially for high or complex fistulas [10]. The robot-assisted technique has gained popularity for its enhanced dexterity, 3D visualization, and precision, particularly in challenging dissections [11].

Key principles of VUF repair include:

- Complete excision of the fistulous tract
- Tension-free multilayered closure of the bladder and uterus
- Interposition of vascularized tissue (typically omentum) to reduce recurrence risk
- Bladder drainage for 10–14 days postoperatively

Our case aligns with these principles.

## Case Report

A 48-year-old para 2 woman underwent a primary cesarean section in 1999, complicated by a postoperative hematoma requiring reoperation. A second cesarean section in 2004 revealed moderate adhesions but was otherwise uneventful.

Seventeen years later, she presented with persistent macroscopic hematuria. Cystoscopy revealed a posterior bladder fistulous opening above the trigone. Methylene blue testing was negative, and MRI confirmed a vesicouterine fistula.

Surgical repair was proposed, initially including hysterectomy, but the patient strongly desired uterine preservation.

A robot-assisted, transperitoneal repair was performed:

- Adhesiolysis and dissection of the vesicouterine space
- Excision of the fistulous tract
- Bladder closure in two layers with barbed sutures
- Uterine closure in two layers
- Omental interposition placed in the vesicouterine fold

Bladder integrity was confirmed intraoperatively. The patient was discharged on postoperative day 1 with a catheter. A cystography at day 14 showed no leakage, and she has remained asymptomatic since.

## Conclusion

This case highlights the feasibility of robot-assisted, uterus-sparing VUF repair via a transperitoneal approach, even in cases with late presentation, decades after cesarean section. Preservation of the uterus may be considered in selected cases, emphasizing the importance of a multidisciplinary approach.

Our findings support the growing body of literature favoring minimally invasive robot-assisted techniques for VUF repair, which offer excellent outcomes with reduced morbidity.

## Supplementary Information

Below is the link to the electronic supplementary material.

Supplementary file1 (MP4 64883 KB)
